# Chk1 and the Host Cell DNA Damage Response as a Potential Antiviral Target in BK Polyomavirus Infection

**DOI:** 10.3390/v13071353

**Published:** 2021-07-13

**Authors:** Lydia E. Hainley, Martina S. Hughson, Amithi Narendran, Ralph Smith, Justin Arthur, Alida Hayner-Buchan, David J. Conti, John M. Lehman, Thomas D. Friedrich

**Affiliations:** 1 Center for Immunology and Microbial Disease, Albany Medical College, Albany, NY 12208, USA; lycivello@gmail.com (L.E.H.); svetlik.martina@gmail.com (M.S.H.); amithi@nycap.rr.com (A.N.); 2 Department of Pathology and Laboratory Medicine, Brody School of Medicine, Greenville, NC 27834, USA; smithra@ecu.edu (R.S.); justingarthur@gmail.com (J.A.); lehmanj@ecu.edu (J.M.L.); 3 Pathology and Laboratory Medicine, Berkshire Medical Center, Pittsfield, MA 01201, USA; ahaynerbuc@bhs1.org; 4 Department of Surgery, Albany Medical College, Albany, NY 12208, USA; contid@amc.edu

**Keywords:** polyomavirus, BKPyV, BKV, DNA damage response, Chk1, antiviral

## Abstract

The human BK polyomavirus (BKPyV) is latent in the kidneys of most adults, but can be reactivated in immunosuppressed states, such as following renal transplantation. If left unchecked, BK polyomavirus nephropathy (PyVAN) and possible graft loss may result from viral destruction of tubular epithelial cells and interstitial fibrosis. When coupled with regular post-transplant screening, immunosuppression reduction has been effective in limiting BKPyV viremia and the development of PyVAN. Antiviral drugs that are safe and effective in combating BKPyV have not been identified but would be a benefit in complementing or replacing immunosuppression reduction. The present study explores inhibition of the host DNA damage response (DDR) as an antiviral strategy. Immunohistochemical and immunofluorescent analyses of PyVAN biopsies provide evidence for stimulation of a DDR in vivo. DDR pathways were also stimulated in vitro following BKPyV infection of low-passage human renal proximal tubule epithelial cells. The role of Chk1, a protein kinase known to be involved in the replication stress-induced DDR, was examined by inhibition with the small molecule LY2603618 and by siRNA-mediated knockdown. Inhibition of Chk1 resulted in decreased replication of BKPyV DNA and viral spread. Activation of mitotic pathways was associated with the reduction in BKPyV replication. Chk1 inhibitors that are found to be safe and effective in clinical trials for cancer should also be evaluated for antiviral activity against BKPyV.

## 1. Introduction

Reactivation of human BK polyomavirus (BKPyV) threatens graft survival in renal transplant recipients. In the absence of a normal immune response in the recipient, BKPyV in the donor kidney can replicate in and destroy the renal tubular epithelium [1]. Serological studies indicate that primary infection by BKPyV occurs early in childhood and that 80–90% of the population is seropositive by adulthood [2,3]. In immunocompetent individuals, a persistent infection is established in epithelial cells of the renourinary tract. It is not known whether a low level of viral replication or transcriptionally inactive viral genomes underlie the persistence [4]. Asymptomatic urinary shedding of BKPyV is present in ~7% of the population [2], increases slightly in immunosuppressed states such as pregnancy [5] and is most pronounced during therapeutic immunosuppression following transplantation [6]. An increase in polyomavirus-associated nephropathy (PyVAN) correlated with the transition from cyclosporine-based therapies to the use of tacrolimus and mycophenolate mofetil in the mid-1990s [7,8]. The progression of PyVAN is generally viewed as a stepwise process starting with viruria in 23–73% of transplant recipients. Appearance of BKPyV DNA in the plasma (DNAemia) is found in 8–62% of transplant recipients, with development of PyVAN in 1–7% of cases [1]. Biopsy-confirmed PyVAN is characterized by expression of virus-encoded large T antigen (TAg), destruction of tubular epithelial cells, interstitial inflammation and fibrosis [9]. BKPyV infection can also result in bladder disease. BKPyV-related hemorrhagic cystitis is a serious complication of allogenic hematopoietic stem cell transplantation [10].

Current success in preventing graft loss from PyVAN depends on regular monitoring of BKPyV viruria and DNAemia coupled with the reduction in immunosuppression, either by decreasing dosage or switching immunosuppressive agents [11,12,13,14,15]. However, when sufficient immunity is restored to inhibit BKPyV replication, there is a corresponding increase in the risk of graft rejection [12,16]. Early detection and response are also critical, because a decrease in BKPyV DNAemia may be delayed as long as 4–10 weeks following reduced immunosuppression [17].

Antiviral drugs that are effective against BKPyV could lessen reliance on immunosuppression reduction, as well as minimize damage from viral replication and acute rejection. Antivirals would also be of special value in cases of PyVAN that do not respond to immunosuppression reduction, or those that evade preemptive screening and present with decreased kidney function. A number of antiviral agents have been used in combination with reduced immunosuppression, but none have demonstrated clear clinical efficacy. Promising results have been reported for cidofovir [18,19,20] and leflunomide [21,22]. Yet, a systematic review of the literature concluded that there was not an obvious benefit to use of these compounds [23]. Brincidofovir, a lipid-conjugated form of cidofovir, is a compound of interest due to improved oral bioavailability and reduced dose-limiting toxicities [24]. It was recently reported that Brincidofovir failed to offer protection from BKPyV disease following allogeneic hematopoietic stem cell transplantation [25]. Fluoroquinolones have also been assessed as anti-BKPyV therapies, but a benefit is not obvious [26]. Intravenous immunoglobulin (IVIG) preparations consist of pooled human immunoglobulin, with high titers of BKPyV-neutralizing antibody. Clearance of viremia and graft survival have been associated with IVIG treatment [27,28]. However, randomized clinical trials are needed to confirm efficacy.

Identification of an antiviral that is safe and effective against BKPyV remains an unmet need in the care of renal transplant recipients. Development of a novel antiviral strategy requires an understanding of the host-pathogen interaction, including the molecular pathways required for BKPyV replication. The BKPyV virion consists of a ~5100 bp circular, nucleosome-packaged DNA surrounded by an icosahedral capsid built of surface-exposed VP1 pentamers and internal pentamer-associated VP2 and VP3 [29]. The viral DNA is functionally divided into three regions: (1) the early gene region encodes the regulatory proteins large T antigen (LTAg), small t antigen (sTAg) and truncated T antigen (truncTAg), which are derived by alternative RNA splicing. Two microRNAs, 3p-miRNA and 5p-miRNA, are encoded in the early region, but expressed from the late promoter on the strand complementary to the TAg mRNAs. Both miRNAs can inhibit TAg expression [30]; (2) the late gene region encodes the capsid proteins VP1, VP2 and VP3, as well as agnoprotein, which disrupts the mitochondrial network, allowing the virus to evade cytosolic innate immune sensing [31]; (3) the noncoding control region (NCCR) includes promoter elements for early and late mRNAs, as well as the origin of viral DNA replication [1,32,33].

BKPyV infection begins with binding of the virion to cell surface gangliosides, primarily GD1b and GT1b [34]. The virion enters monkey kidney cells by caveolin-dependent endocytosis [35], whereas entry into human renal proximal tubule epithelial cells is through a clathrin and caveolin-independent mechanism [36]. Endosome trafficking to the endoplasmic reticulum (ER) depends on an intact microtubular network [37]. Within the ER, the virion partially disassembles and is retrotranslocated into the cytosol via the endoplasmic reticulum-associated degradation (ERAD) pathway [38]. Nuclear location sequences in VP2 and VP3, which are exposed by the partial disassembly, target the virion to the nuclear pore [39]. After arrival of the viral DNA in the cell nucleus, transcription factor binding to the NCCR drives expression of early mRNAs, which are translated into the TAg proteins. Quiescent cells are induced to enter S phase by the binding of LTAg to retinoblastoma protein family members and p53, and sTAg binding to the phosphatase PP2A. Once in S phase, two LTAg hexamers assemble on the origin of replication and locally unwind the viral DNA. Subsequent steps in bidirectional viral DNA replication are carried out by host cell proteins. After viral DNA replication is initiated, changes in transcription factor binding to promoter elements result in decreased expression of early mRNAs and increased expression of late mRNAs and viral capsid proteins. Virions are assembled in the nucleus and appear to be released by cell lysis [40,41].

Novel antiviral approaches that target early events in BKPyV infection are being explored in cell culture models. Glibenclamide, a channel blocker that is clinically approved for treatment of diabetes mellitus type 2, inhibits trafficking of BKPyV to the endoplasmic reticulum. Inhibition of the cystic fibrosis transmembrane conductance regulator by glibenclamide is an essential component of the mechanism of action [42]. Inhibition of BKPyV infection can also be achieved with a 13 amino acid peptide derived from BKPyV VP2/3. The peptide binds to the upper VP1 pentamer pore and appears to inhibit an early step after binding of the virion to the cell surface and before retrotranslocation from the endoplasmic reticulum to the cytosol [43].

Multiple polyomaviruses have been shown to induce a host cell DNA damage response (DDR) and several are reported to depend on the DDR for optimal virus replication [44,45,46,47,48]. The DDR includes two arms controlled by the protein kinases ATM and ATR [49]. ATM is best known for its response to double strand breaks and activation of the protein kinase Chk2 through phosphorylation. ATR primarily responds to regions of single strand DNA and activates the protein kinase Chk1 through phosphorylation. Chk1 and Chk2 phosphorylate substrate proteins that arrest the cell cycle and participate in DNA repair. H2AX is a subtype of histone H2A that is termed γH2AX when phosphorylated at Ser139. The protein kinases ATM, ATR and DNA-PK are all capable of phosphorylating H2AX Ser139 in response to DNA damage [50]. H2AX is phosphorylated in response to in vitro infection by the polyomaviruses SV40 [46], JCPyV [51], MCPyV [52], MuPyV [53] and BKPyV [48]. Other DNA viruses including B19 parvovirus [54], KSHV [55,56,57], HSV-1 [58], cytomegalovirus [59], adenovirus [60] and hepatitis B virus [61] are also known to stimulate phosphorylation of H2AX.

Recent reports describe the impact of ATM and ATR inhibition on BKPyV replication and the host cell cycle [48,62]. Stimulation of ATR kinase activity by BKPyV infection prevents the onset of mitosis and extends S phase, allowing for continued viral DNA replication [62]. Inhibition of ATR kinase activity in BKPyV-infected RPTECs results in the initiation of mitotic pathways in S phase cells. Small molecule inhibitors of the ATR substrate, Chk1, have been shown to decrease replication of both JCPyV [45] and BKPyV [48].

Considering that Chk1 inhibitors are being evaluated in clinical trials as anticancer agents, it may be possible to repurpose Chk1 inhibitors for the treatment of BKPyV disease. We explored the antiviral effects of Rabusertib (LY2603618), the focus of multiple clinical trials against different forms of cancer [63,64,65,66,67,68,69]. LY2603618 did not progress beyond phase II, but is recognized as the most specific Chk1 inhibitor. We hypothesized that treatment of BKPyV-infected cells with LY2603618 would result in the inhibition of Chk1 activity and BKPyV replication. Our results demonstrate that concentrations of LY2603618 that inhibited Chk1 autophosphorylation also limited the replication of BKPyV DNA and reduced the progression of infected cells into >G2 phase. siRNA-mediated knockdown of Chk1 confirmed that loss of Chk1 resulted in decreased replication of BKPyV DNA. Both LY2603618 and Chk1 siRNA also increased phosphorylation of histone H3 ser10 (pH3-S10) confirming that Chk1, like ATR, is essential for maintaining an S phase environment to support BKPyV DNA replication.

## 2. Materials and Methods

### 2.1. Cells and Virus

Human proximal tubule epithelial cells (RPTECs, #CC-2553) and renal epithelial cell growth medium (REGM, #CC-3190) were obtained from Lonza. The cell population was expanded through four population doublings in fully supplemented REGM, aliquoted and frozen in liquid nitrogen. Viral stocks were obtained by infecting T75 flasks of low passage, near-confluent RPTECs with either the Gardner strain (American Type Culture Collection, VR-837) or Dunlop strain of BKPyV at an MOI of 0.01 IU/cell. Dunlop virus stocks were initiated from transfection of RPTEC with viral DNA excised from pBKV (American Type Culture Collection, 34-2) as described [70]. Culture medium was changed every 4 days for a period of 3 weeks until a cytopathic effect was obvious in a large percentage of the population. The cells were subjected to 6 cycles of freeze–thaw and then sonicated to release cell-associated virus. Cell debris were removed by centrifugation at 1500× *g* for 10 min and the supernatant was aliquoted and stored at −80 °C. BKPyV stocks were titered by endpoint dilution in RPTECs; titers of 1 to 3 × 10^8^ infectious units (IU) per ml were obtained. When compared by laser scanning cytometry (LSC) for expression of TAg and DNA content, the Dunlop-infected cells exhibited a tighter range of TAg expression (Figure S1). This difference most likely results from a comparatively lower genetic heterogeneity of the newly established Dunlop virus stock, compared to the Gardner virus stock, which has been maintained in vitro for years.

RPTEC cultures were established by plating 1 × 10^6^–1.5 × 10^6^ cells in REGM in 60 mm plates. The medium was replaced 24 h later. Once cells reached near confluence, approximately 72 h later, the medium was replaced with renal epithelial cell basal medium (REBM, #CC-3191, Lonza, Basel, Switzerland), supplemented with 0.5% fetal bovine serum plus 30 µg/mL gentamycin/15 ng/mL amphotericin, for 48 h to achieve quiescence.

LY2603618 (S2626, Selleck Chemicals, Houston, TX, USA) was prepared as a 20 mM stock in DMSO, aliquoted and stored at −20 °C. In all experiments involving treatment with different concentrations of LY2603618, the DMSO is held at the same concentration in all LY2603618 dilutions.

### 2.2. Immunohistochemical and Immunofluorescent Analysis of Biopsies

Kidneys of transplant recipients were biopsied by the Albany Medical Center (AMC) Division of Renal and Pancreatic Transplant Services. Nine archival PyVAN biopsies and sections of normal kidney were provided by the AMC Department of Pathology and Laboratory Medicine. The study protocol was approved by the AMC Institutional Review Board. Sections of formalin-fixed, paraffin-embedded PyVAN biopsies were deparaffinized, rehydrated and subjected to antigen retrieval in Citrate Buffer (Thermo Fisher, Waltham, MA, USA) for 60 min in a food steamer. Endogenous peroxidase activity was blocked by treatment with 0.3% hydrogen peroxide in PBS. A 20 min incubation in blocking buffer, consisting of either 0.15% normal goat serum (NGS) or normal horse serum (NHS) in PBS, was followed by overnight incubation at 4 °C with primary antibody against SV40 TAg (1:40, DP02 Calbiochem, Burlington, MA, USA) or pH2AX-Ser139 (1:200, 20E3, Cell Signaling, Danvers, MA, USA) in NGS or NHS blocking buffer, respectively. Slides were rinsed in PBS and incubated with biotinylated secondary antibody in the appropriate blocking buffer for 60 min at room temperature. Slides were rinsed in PBS and incubated with preformed avidin:biotin enzyme complex (Vectastain Elite, Vector labs, Burlingame, CA, USA) followed by incubation with DAB substrate. Sections were counterstained with 0.5% methyl green in 0.1 M sodium acetate buffer, pH4.2 prior to dehydration. Hematoxylin and eosin (H&E)-stained sections [71] were used for histological PyVAN staging.

For dual immunofluorescent analysis, tissue sections were incubated with NGS blocking buffer, followed by overnight incubation at 4 °C in a mixture of 1:40 SV40 TAg (PAb416) and 1:200 pH2AX-Ser139 antibodies, rinsed in PBS and incubated with a mixture of Alexa488-conjugated goat anti-mouse (1:200) and Alexa594-conjugated goat anti-rabbit (1:200) antibodies in NGS blocking buffer. Slides were rinsed in PBS and mounted in 90% glycerol/0.5% N-propyl gallate in PBS. Sections were viewed with an Olympus FlouView 1000 confocal microscope using a 60× water/oil immersion objective. Alexa 488 and 594 were visualized with 488 nm and 559 nm excitation lasers using 505–540 and 575–675 band pass filters. Data were collected sequentially from each channel at a resolution of 512 × 512 pixels.

### 2.3. Immunofluorescent Staining of Cultured Cells

RPTECs were grown on glass cover slips that were pre-coated overnight with 10% fetal bovine serum in PBS to improve cell attachment. Cells were fixed by removing media, rinsing with PBS and adding 100% methanol (−20 °C) to the dish for 10 min. Fixed cover slips were air dried and stored at −20 °C. Fixed cells were rehydrated in PBS, incubated with primary antibody in 1% BSA/PBS or NGS blocking buffer for 1 h, washed 5X with PBS and incubated with Alexa-conjugated secondary antibody for one hour. After washing 5X with PBS, the cover slips were quickly rinsed with dH_2_0 and mounted in PBS/glycerol. Primary antibodies used for immunofluorescence were as follows: mouse anti-SV40 TAg (PAb416, Calbiochem), mouse anti SV40 VP1 (PAb597, a gift from Ed Harlow) and rabbit anti-pH2AX-Ser139 (20E3, Cell Signaling). To measure percent TAg-positive cells, cover slips were stained with 300 nM DAPI for 30 min and analyzed with Cell Profiler software.

### 2.4. Laser Scanning Cytometry (LSC)

Simultaneous quantification of antibody staining (TAg, VP1 or γH2AX) and cell DNA content was carried out as previously described [72]. Briefly, antibody staining of cell monolayers was carried out as described in Section 2.3. Stained cell monolayers on cover slips were then incubated with 500 μg/mL RNase A, 50 μg/mL propidium iodide (PI) at 37 °C for 30 min and mounted in 90% glycerol/10% PBS containing 100 μg/mL PI. Data were acquired with an iCys research laser scanning cytometer (Compucyte, Cambridge, MA, USA; now Thorlabs, Newton, NJ, USA). Cytometric Analysis Software version 3.2.5 (iGeneration) was used for data acquisition and analysis. Instrument settings were 5 mW laser power (488 nm argon) with detector voltages set at 35 V green channel with a 530/30 optical filter and 33 V long red channel with a 650 LP optical filter. For reanalysis, the contour primary minimum area of 20 µm^2^ was set to exclude cell fragments and a maximum area of 340 µm^2^ to allow doublet discrimination. The instrument was set to acquire data on 5000 cells per sample and the following parameters on each cell were collected and stored: these included red fluorescence (DNA [PI]), green fluorescence (TAg, VP1 or γH2AX) and the cell coordinates (location). The data were first displayed as a plot of red fluorescence per cell (DNA, in linear scale on the *x* axis) vs. the number of cells (count) in that channel on the *y* axis also in a linear scale to determine cell cycle position. The panels on the right of Figure S2 are a representation of this initial data plot without the coloring of the cell cycle phases. The left-hand panels of Figure S2 are the plots of the DNA content (*x* axis) vs. the antigen fluorescence in log on the *y* axis but not gated (colored). G1, S, G2 and >G2 phases were gated, based on the G1 and G2 phase peaks of uninfected cells, and colored to represent the cells position in the cell cycle (cyan for G1 phase cells, black for S phase cells, red for G2 phase cells and blue for >G2 cells. The data were then displayed as dot plots of DNA per cell (*x* axis, linear scale, “Long Red Integral”) and green fluorescence (TAg, VP1 or γH2AX) per cell (*y* axis, log scale, “Green Integral”). To compare positive and negative antigen-expressing cells (TAg, VP1 or γH2AX), gates were placed on the positive, expressing cells (R5) and non-expressing cells (R4). This was the format of presentation in all LSC dot plots. Cells with extremely low levels of green fluorescence were present in the R7 gate at the very bottom of scatter plots. The vast majority of these cells had a G1/G0 phase, 2C DNA content regardless of the number of days post-infection, consistent with the absence of viral protein expression or cell cycle progression (Figure S2, right-hand panels).

### 2.5. Immunoblotting

Cell monolayers were rinsed with PBS and directly lysed in SDS-PAGE sample buffer at 1 × 10^7^ cells/mL. Cells floating in culture medium were collected by centrifugation and pooled with the monolayer lysates. Prior to electrophoresis, lysates were sonicated to fragment DNA and heated at 95 °C for 5 min. Lysates were resolved by electrophoresis on precast 4–20% or Any KD Mini-Protean gels (BioRad, Hercules, CA, USA) or 3–8% Tris-Acetate gels (NuPAGE Novex, Carlsbad, CA, USA) and wet-transferred to PDVF membranes. Blocking protocols and antibody dilutions recommendations from the manufacturer and specific to the primary antibody were followed. All primary antibody incubations were overnight on a shaker at 4 °C. Blots were rinsed 3X for 10 min each in TTBS (20 mM Tris-HCl, 150 mM NaCl, 0.05% Tween-20, pH 7.5) and incubated with HRP-conjugated goat anti-mouse or goat anti-rabbit IgG on a shaker at room temperature for 1 h. After 3X 10 min rinses in TTBS, blots were incubated with either SuperSignal^®^ West FEMTO or PICO. Bands were visualized using X-ray film or imaging (BioRad ChemiDoc) and quantified using ImageJ. Blots were stripped for reprobing with 6 M guanidine HCl, 20 mM Tris HCl pH7.5, 0.2% NP-40, 0.7% β-mercaptoethanol [73]. The following antibodies were used for immunoblotting: TAg-PAb416 (#DP02, Calbiochem and Millipore Sigma); VP1-PAb 597 (isolated from hybridoma); lamin A/C (#612163, BD Transduction Laboratories, Franklin Lakes, NJ, USA); pH3-Ser10 (#Q16695, Millipore). From Abcam: ATM (#ab82512) and pATM-Ser1810 (#ab81292). From Cell Signaling: pChk1-Ser296 (#2349), pChk1-Ser317 (#2344), pChk1-Ser345 (#2341), Chk1 (#2360), pChk2-Thr68 (#2197), Chk2 (#3440), H2AX (#9718), and Histone 3 (#9715). From Santa Cruz Biotechnology (Dallas, TX, USA): GAPDH (#sc25778), goat anti-rabbit IgG-HRP (sc-2004), and goat anti-mouse IgG-HRP (sc-2005).

### 2.6. Alamar Blue Assay

RPTECs were plated in 96-well tissue culture plates at 1.5 × 10^3^ cells/well in 200 µL complete REGM. Cells were brought to quiescence as described in Section 2.1. LY2603618 (20 mM in DMSO) was diluted in REBM/0.5% FBS, with 0.08% DMSO in all dilutions. After 3 days of treatment, 20 µL of Alamar Blue was added to each well and the cells were returned to the 37 °C incubator for 9 h. Cell viability was quantified by measuring the absorbance of the oxidized (600 nm) and reduced (570 nm) forms of Alamar Blue and calculating the percentage reduction.

### 2.7. siRNA-Mediated Knockdown

RPTECs were plated and grown to near confluence in complete REGM. Culture medium was then replaced with REBM/0.5% FBS for two days. RNAi duplex-Lipofectamine RNAiMAX complexes were made by first preparing 60 nM solutions of the following RNAi duplexes (all from Ambion): Chk1 (# 4390824), GAPDH (# 4390849), negative control #1 siRNA (# 4390843) in Opti-MEM I Reduced Serum. Lipofectamine RNAiMAX was diluted 1:50 in Opti-MEM Reduced Serum Medium. The diluted RNAi duplexes were combined with the diluted Lipofectamine RNAiMAX and incubated for 20 min at room temperature. Medium was removed from RPTEC cultures and replaced with Opti-MEM Reduced Serum Medium. The RNA-duplex-Lipofectomine RNAiMAX complexes were then added to a final siRNA concentration of 5 nM. The plates were gently rocked and placed in a 37 °C CO_2_ incubator. Following a 24 h treatment with siRNA, the complexes were removed and RPTECs were infected with BKPyV (MOI = 10) in Opti-MEM Reduced medium for 90 min at 37 °C in a CO_2_ incubator. The virus inoculum was removed and replaced with Opti-MEM Reduced Medium. Plates were placed in 37 °C, 5% CO_2_ incubator for 72 h. Parallel cell cultures were either trypsinized and pelleted for viral DNA analysis or directly lysed in 1× sample buffer for immunoblotting.

### 2.8. Quantification of Viral DNA

#### 2.8.1. DNA Isolation from Cells and Electrophoresis

Floating cells were collected by centrifugation, combined with trypsinized monolayers, pelleted and stored at −20 °C. BKPyV DNA was isolated from cell pellets using a modification of the QIAprep Spin Miniprep Kit as described by Ziegler et al. [74]. Total cell DNA was purified from cell pellets using the Qiagen DNeasy blood and tissue kit. Purified DNA was linearized with Kpn1 or BamH1 prior to electrophoresis on a 0.6% agarose gel. Gels were loaded with equal volumes of QIAprep eluate or equal amounts of DNA from DNeasy spin columns. Ethidium bromide-stained gels were imaged and band intensity was quantified with Image J.

#### 2.8.2. Real-Time PCR

Culture medium was removed from plates, clarified at 5000× *g* for 15 min at 4 °C and stored at −20 °C. Qiagen DNeasy blood and tissue mini-spin columns were used to isolate DNA from 200 µL of thawed culture media. Equal volumes of purified DNA were amplified in triplicate with an Applied Biosystems StepOnePlus real-time PCR system. pBKV 34-2 DNA was used for the standard curve. The BKPyV primers were as described [75]:

Forward primer: AGCAGGCAAGGGTTCTATTACTAAAT

Reverse primer: GAAGCAACAGCAGATTCTCAACA

Amplifications were carried out in 20 µL volumes including 5 µL of DNA sample, 100 nM forward and reverse primers in SYBR Select Master Mix (#4472908, Applied Biosystems). The temperature cycle was initiated with a 2 min hold at 50 °C, followed by a 10 min hold at 95 °C and 45 cycles of 95 °C for 15 s and 60 °C for 60 s. StepOne software version 2.2.2 was used for data analysis.

## 3. Results

### 3.1. DDR Activation in BKPyV-Infected Cells in PyVAN

**Figure 1:**
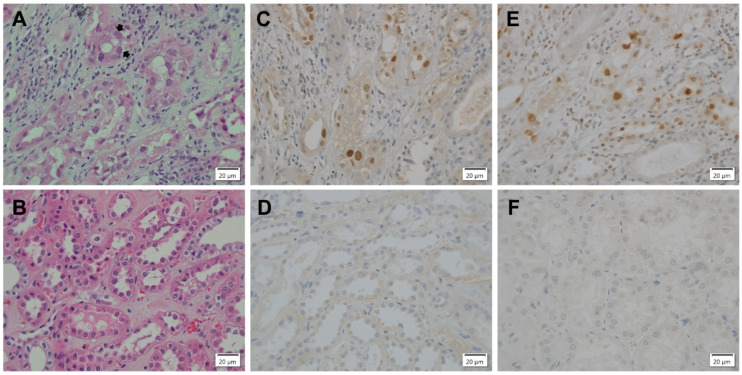
IHC detection of a DNA damage response in PyVAN. Formalin-fixed, paraffin-embedded PyVAN biopsy, stage B2 (**A**,**C**,**E**) and normal kidney (**B**,**D**,**F**). (**A**) Interstitial inflammation with edema and fibrosis. Marked viral activation with tubular epithelial cell lysis and associated denudation of tubular basement membranes. Arrows indicate amorphous, ground glass polyomavirus inclusion bodies (H&E stain). (**B**) Normal kidney (H&E stain). (**C**) IHC detection of intranuclear polyomavirus TAg in tubular epithelial cells. Positive nuclei contain brown diaminobenzadine reaction product (PAb 416, anti-SV40 TAg primary antibody). (**D**) Normal kidney stained for TAg, as in C. (**E**) IHC detection of DDR marker γH2AX (anti pH2AX-Ser139 primary antibody) in nuclei of tubular epithelial cells. (**F**) IHC normal kidney stained for γH2AX as in E. (40X objective).

To determine whether BKPyV infection of renal tubular epithelium in vivo is associated with the activation of a DDR, archival PyVAN biopsies were immunohistochemically (IHC) stained for γH2AX and BKPyV T antigen (TAg). PyVAN biopsies were examined by H&E staining and ranked as either histological stage A, B or C [76,77,78]. A representative H&E-stained stage B2 biopsy exhibiting typical features of PyVAN including epithelial cell lysis and nuclear viral inclusions (arrows) is shown in Figure 1A. Neighboring sections were IHC stained with antibody against TAg or γH2AX. The nuclei of some TAg-expressing cells (Figure 1C) were enlarged relative to those of uninfected cells (Figure 1D) as previously noted by others [79]. IHC staining for γH2AX was also observed in nuclei of tubular epithelial cells indicating activation of a DDR (Figure 1E). Similar IHC results were found in all PyVAN biopsies, regardless of histological stage. Cells in sections of normal kidney did not stain positive for TAg (Figure 1D) or γH2AX (Figure 1F).

**Figure 2:**
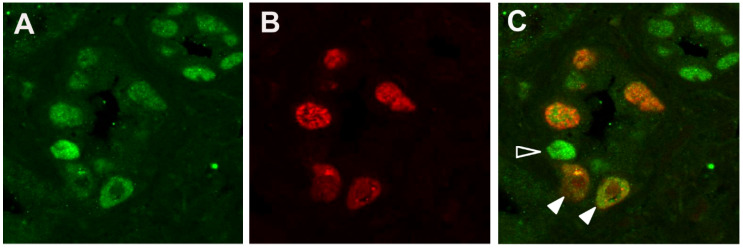
Dual immunofluorescent detection of a DNA damage response in virus-infected cells in PyVAN. Formalin-fixed paraffin-embedded PyVAN biopsy (stage B2). (**A**) Polyomavirus-infected tubular epithelial cells visualized by immunofluorescent staining for TAg (PAb416 and Alexa488 goat anti-mouse IgG). (**B**) Intranuclear γH2AX in tubular epithelial cells (anti pH2AX-Ser139 and Alexa594 goat anti-rabbit IgG). (**C**) Merge of images in A and B. Open arrowhead indicates a TAg-positive, γH2AX-negative tubular epithelial cell; filled arrowheads indicate TAg-positive, γH2AX-positive cells with nuclear inclusions. (60× objective).

The presence of both TAg-positive cells and γH2AX-positive cells within the tubular epithelium led us to ask whether phosphorylation of H2AX was occurring in BKPyV-infected epithelial cells. To answer this question, PyVAN biopsies were analyzed by immunofluorescent staining. A stage B2 biopsy from another patient was co-stained and images for TAg (Figure 2A) and γH2AX (Figure 2B) were merged (Figure 2C). All nuclei in the large tubule (center of the image) that were positive for γH2AX also expressed TAg, demonstrating that phosphorylation of H2AX is occurring in BKPyV-infected cells. However, not all TAg-expressing cells were positive for γH2AX. The bright TAg-positive nucleus (open arrowhead, Figure 2C) in the large tubule and all TAg-positive nuclei in the smaller tubule (upper right) lack γH2AX, indicating that γH2AX is present in a subpopulation of Tag-expressing cells. The presence of γH2AX in TAg-positive nuclei bearing inclusions (solid arrowheads, Figure 2C) shows that γH2AX can be present in late stages of the virus life cycle.

### 3.2. BKPyV Infection Stimulates DNA Synthesis beyond 4C in Cultured Human RPTECs

**Figure 3:**
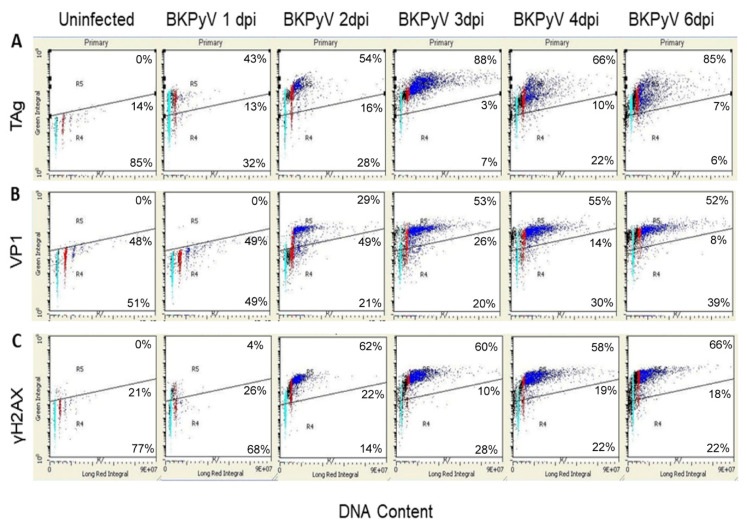

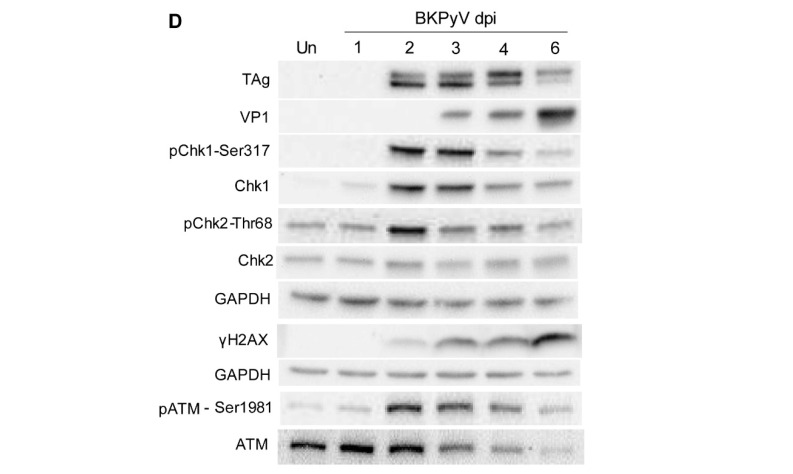
BKPyV infection of RPTECs stimulates a DDR and continued DNA synthesis beyond the normal G2 phase DNA content. Quiescent RPTECs on cover slips were infected with BKPyV Gardner (MOI = 10 IU/cell) and methanol fixed at the indicated days post-infection (dpi); uninfected cells at 0 dpi (Un). (**A**) Cells were stained for TAg (PAb416 and Alexa 448 goat anti-mouse IgG), (**B**) VP1 (PAb597 and Alexa 488 goat anti-mouse IgG) or (**C**) γH2AX (anti pH2AX-Ser139 and Alexa 488 goat anti-rabbit IgG). All cover slips were treated with RNaseA and stained with propidium iodide prior to mounting. Alexa488 and PI were quantified by laser scanning cytometry. Cell cycle position is color coded as follows: G1 phase (cyan), G2 phase (red) and ˃G2 phase (blue). Sub-G1 and S phase cells are represented in black. The angled horizontal line indicates the division between positive and negative cell populations. Percentages along right side of plots indicate the percentage of the cell population in gates R5, R4, R7 (moving from top to bottom). (**D**) Whole-cell lysates were prepared from pooled floating and adherent cells by lysing in sample buffer, resolving by SDS PAGE and immunoblotting with the indicated antibodies. Membranes were first probed for phosphoproteins, stripped and then probed with antibody recognizing both phosphorylated and unphosphorylated forms. After incubation with either HRP-conjugated goat anti-mouse or goat anti-rabbit IgG, blots were incubated with SuperSignal West and imaged. TAg, VP1, Chk1, Chk2 and the upper GAPDH were analyzed on the same 4–20% gel; γH2AX and the lower GAPDH on an AnyKD gel; ATM on a 3–8% gel. The change in the ratio of the doublet bands of TAg (large T) between 3 and 4 dpi was not consistently observed; representative blot of *n* = 3.

To better define the role of the DDR in BKPyV infection, RPTECs were infected with BKPyV in vitro. Virus infection was analyzed by two-color LSC for single-cell measurement of BKPyV TAg, or viral capsid protein VP1, and total cellular DNA content. Quiescent RPTECs were infected with BKPyV at 10 infectious units (IU) per cell. Quiescent, rather than proliferating cultures, were used to test the full range of viral functions in stimulating cell cycle progression and to provide a more synchronous population for analysis. TAg was expressed in G1, S and G2 phase cells at 1 day post-infection (dpi). The percentage of TAg-expressing cells and the quantity of TAg per cell increased over the first 3 dpi (Figure 3A). TAg-expressing cells did not exhibit the normal cell cycle behavior of progression through S, G2 and M phases. DNA synthesis continued beyond the normal 4C DNA content of G2 phase cells, generating a population of >G2 phase cells (Figure S3A). At 3 dpi, >G2 phase infected cells had DNA contents between 4C and 14C (Figure 3A). Expression of the viral coat protein VP1 exhibited a distinct cell cycle-dependent pattern (Figure 3B). Unlike TAg, which was initially expressed in G1 and S phase cells, VP1 expression was delayed and first appeared in the G2 and >G2 phase populations. This is consistent with the previously described increase in late mRNA and VP1 expression following the onset of polyomavirus DNA synthesis [80].

### 3.3. BKPyV Infection Stimulates a DDR in Cultured Human RPTECs

The presence of γH2AX in PyVAN biopsies led us to investigate the BKPyV-induced DDR in vitro. By LSC analysis, there was a small increase in γH2AX-positive S phase cells at 1 dpi (Figure 3C). By 2 dpi, infected cells progressed into >G2 phase and expressed significantly higher levels of γH2AX (Figure 3C). The cell cycle-associated pattern of γH2AX expression more closely matched that of TAg (Figure 3A) than VP1 (Figure 3B).

Protein expression and phosphorylation were also monitored by immunoblot analysis. TAg was first detected at 2 dpi and continued to be expressed for the remainder of the time course, decreasing at 6 dpi (Figure 3D). The capsid protein VP1 increased with delayed kinetics relative to TAg and maintained a high level until 6 dpi. The onset of viral protein expression at 2 dpi coincided with the synthesis and/or activation of protein kinases in DDR pathways. Chk1 was present at very low levels in resting cells but increased in response to viral infection (Figure S3B). ATM and Chk2 were expressed in uninfected quiescent cells. At 2 dpi, all three protein kinases were phosphorylated at sites known to be associated with activation. Phosphorylation of Chk1 at S317 and S345, two sites known to be phosphorylated by ATR [81], indicated activation of the ATR–Chk1 pathway. In addition, activation of the ATM pathway was evident by phosphorylation of ATM S1981 and the ATM substrate, Chk2 T68. ATM is recruited to dsDNA breaks where the inactive dimer is converted to active monomers by autophosphorylation at S1981 [82]. ATM S1981 can also be phosphorylated by ATR activated by replication fork stalling or UV treatment [83]. The kinetics of H2AX phosphorylation also paralleled viral protein expression and activation of Chk1 and Chk2. Levels of TAg and the DDR kinases decreased late in infection, whereas VP1 and γH2AX were maintained or increased (Figure 3D).

### 3.4. Inhibition of Chk1 by LY2603618 in BKPyV-Infected Cells

**Figure 4:**
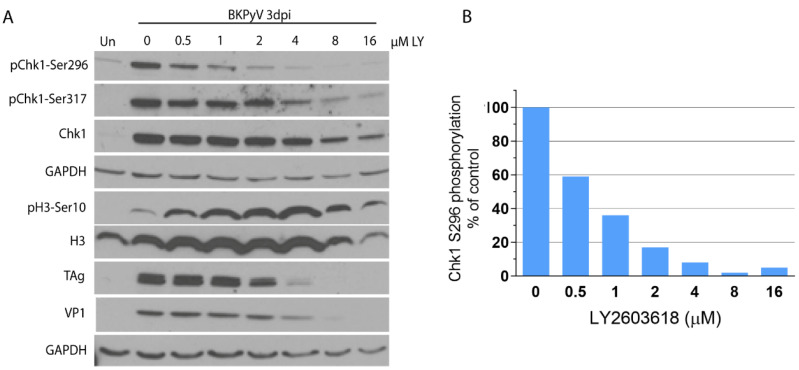
Concentration-dependent effects of LY2603618 on Chk1 autophosphorylation and viral protein expression. Quiescent RPTECs were infected with BKPyV Gardner (MOI = 10 IU/cell) for 1.5 h and incubated in the indicated LY2603618 concentrations for three days. Uninfected cells (Un) serve as a negative control. (**A**) Cells were harvested at 3 dpi by lysis with SDS-PAGE sample buffer and analyzed by immunoblotting with the indicated antibodies; representative of *n* = 4. (**B**) Band intensity of pChk1-Ser296, the Chk1 autophosphorylation site, was normalized to intensity of the total Chk1 band and presented as percent of untreated, BKPyV-infected control. An IC50 = 640 nM was calculated with GraphPad prism.

Clinically-relevant inhibitors of enzymes in DDR pathways may be valuable both for preventing kidney damage due to BKPyV replication and for gaining a mechanistic understanding of the DDR in BKPyV infection. Ongoing clinical trials of Chk1 inhibitors as anticancer agents [84] led us to examine a clinically-tested Chk1 inhibitor for its effect on BKPyV infection in vitro. The Chk1 inhibitor LY2603618 (Rabusertib) has been evaluated in at least seven phase I/II clinical trials [85] and is the most specific Chk1 inhibitor [86]. To determine whether this inhibitor could effectively inhibit Chk1 activity in our system, BKPyV-infected RPTECs were treated with a concentration range of LY2603618 for 3 days. Autophosphorylation of Chk1-Ser296 served as the primary indicator of Chk1 activity. As shown in Figure 4A,B, a concentration-dependent inhibition of Chk1-Ser296 phosphorylation was observed with an IC50 of 640 nM and greater than 90% Chk1 inhibition at 4 µM and above.

It has been reported that Chk1 inhibition by LY2603618 can override the G2/M phase checkpoint in HeLa cells and initiate premature mitotic events [87]. To determine whether Chk1 inhibition initiated mitotic events in the context of virus infection, the mitotic marker, pH3-Ser10, was monitored by immunoblot analysis. Correlation between the concentration-dependent decrease in Chk1-Ser296 autophosphorylation and the increase in pH3-Ser10 was consistent with initiation of mitotic events due to lost checkpoint control. LY2603618 concentrations that inhibited Chk1 activity by 85% or more were also associated with decreased expression of the virus-encoded proteins large T (TAg) and VP1 (Figure 4A). Concentrations of LY2603618 that inhibited Chk1 activity by 50–90% had a slight effect on the viability of uninfected cells, with greater toxicity at higher concentrations (Figure S4).

### 3.5. LY2603618 Restricts Progression of BKPyV-Infected Cells into >G2 Phase

**Figure 5:**
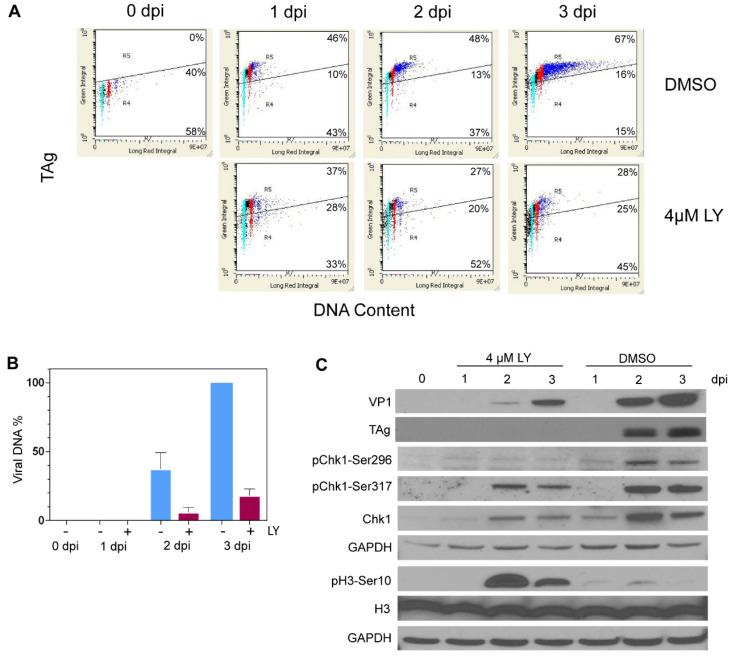
LY2603618 inhibits BKPyV DNA synthesis and progression of infected cells into >G2 phase. Quiescent RPTECs were infected with BKPyV (MOI = 10 IU/cell) for 90 min. The inoculum was removed and either 4 µM LY2603618 or 0.025% DMSO was added to parallel cultures. (**A**) Cultures were fixed on days 0 (uninfected), 1,2 and 3 post-infection. Fixed cells were stained with anti-SV40 TAg (PAb416) followed by Alexa 488 goat anti-mouse IgG. Cells were treated with RNase A and stained with propidium iodide prior to LSC analysis. Cell cycle position is color coded as follows: G1 phase—cyan, S phase—black, G2 phase—red and >G2 phase—blue. (**B**) Viral DNA was isolated from pooled floating and adherent cells using Qiagen miniprep spin-columns. The DNA was then linearized and resolved on ethidium bromide-containing agarose gels, imaged and quantified with Image J; DMSO (blue), LY2603618 (magenta); *n* = 3, error bars indicate standard error of the mean. (**C**) Whole-cell lysates were prepared from pooled floating and adherent cells by lysing in SDS-Page sample buffer and immunoblotting with the indicated antibodies; representative of *n* = 3.

To assess the impact of LY2603618 on the progression of BKPyV infection, parallel cultures of quiescent RPTECs were treated with 4 µM LY2603618 beginning at 1.5 h post-infection (hpi). Cultures were then harvested for LSC, immunoblot and viral DNA analysis, each day until 3 dpi. Cytometric analysis showed that LY2603618-treatment limited progression of infected cells into >G2 at 2–3 dpi (Figure 5A and Figure S5A). The TAg mean fluorescent intensity (MFI) of TAg-positive cells increased over time in control cells, but plateaued in LY2603618-treated cells (Figure S5B).

**Figure 6:**
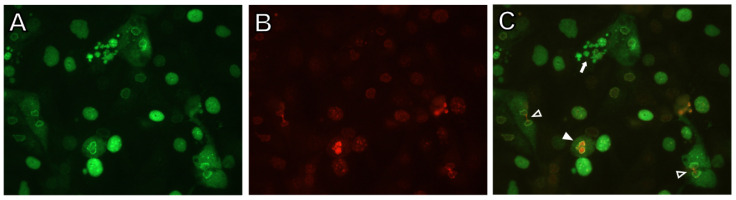
Abnormal mitotic events in LY2603618-treated BKPyV-infected cells. Quiescent RPTECs were infected with BKPyV Gardner (MOI = 10 IU/cell) for 1.5 h. The inoculum was removed and culture medium containing 4 µM LY2603618 DMSO was added. Cells were fixed at 3 dpi. Cells were first incubated with combined primary antibodies, rinsed and then incubated with combined secondary antibodies. (**A**) Anti-TAg (Pab416) and Alexa 488 goat anti-mouse IgG; (**B**) anti-pH3-Ser10 and Alexa 594 goat anti rabbit IgG; (**C**) merged TAg and pH3-Ser10 images. Open arrowheads: pH3-Ser10-stained intercellular bridges in telophase cells; filled arrowhead: A single cell containing two connected TAg and pH3-Ser10-stained chromatin masses; filled arrow: TAg-stained fragmented nuclei. (40× objective).

Consistent with the decrease in the >G2 population, replication of viral DNA in LY2603618-treated cultures was reduced by 80–85% at 2–3 dpi relative to vehicle-treated control cultures (Figure 5B and Figure S6). Immunoblot analysis revealed that Chk1 levels were very low in quiescent cultures and increased beginning at 1 dpi (Figure 5C). Active Chk1, detected by pChk1-Ser296 staining, was significantly decreased in LY2603618-treated cells, indicating efficient inhibition of Chk1 activity. Expression of BKPyV TAg in treated cells was also reduced and did not attain the level seen in vehicle-treated cells (Figure 5C). Increased phosphorylation of H3-Ser10 after 2–3 dpi in the presence of LY2603618 indicated activation of mitotic pathways. The initiation of mitotic events in LY2603618-treated BKPyV-infected cells was confirmed by immunofluorescent microscopy. Cells were fixed at 3 dpi and stained for both TAg and the mitotic marker pH3-Ser10 (Figure 6). A subpopulation of TAg-positive cells was in the late stages of mitosis. Telophase cells exhibited both diffuse cytoplasmic TAg and chromatin-associated TAg; some with intercellular bridges positive for pH3-Ser10 (open arrowhead). TAg-positive fragmented nuclei were also present (filled arrow).

The impact of LY2603618 treatment on total cellular DNA synthesis was examined by pulsing with the thymidine analogue 5-ethynyl-2″-deoxyuridine (EdU) for one hour prior to fixation at 3 dpi, followed by LSC analysis (Figure S6). In untreated BKPyV-infected cultures, DNA synthesis was ongoing in cells from S phase through >G2 phase. In virus-infected cultures treated with LY2603618, cells with S, G2, and early >G2 phase DNA contents were synthesizing DNA, but there was a reduction in the number of labeled cells at higher DNA contents.

### 3.6. LY2603618 Reduces BKPyV Spread In Vitro

**Figure 7:**
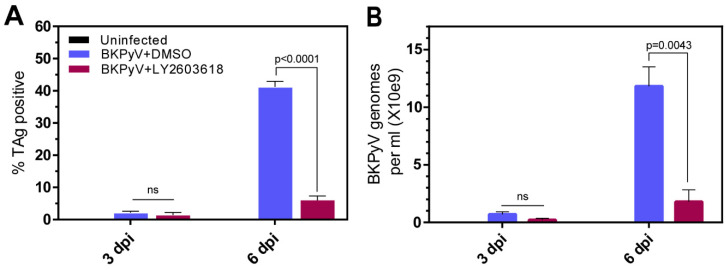
LY2603618 limits spreading BKPyV infection in vitro. Parallel cultures of quiescent RPTECs were infected with BKPyV Dunlop (MOI = 0.1 IU/cell) and treated with either 4 µM LY2603618 or 0.02% DMSO beginning at 1.5 hpi. Cells and culture media were harvested at 3 dpi and 6 dpi. For cultures harvested at 6 dpi, media including LY2603618 or DMSO was replaced at 3 dpi. (**A**) Percent TAg-positive cells. Cells on cover slips were fixed, stained with anti-TAg (PAb416), followed by goat anti-mouse Alexa 594. Cover slips were stained with DAPI prior to immunofluorescent microscopy and quantification of percent TAg-positive cells by Cell Profiler Count and Score software. (**B**) BKPyV DNA release into culture media. DNA was purified from culture media and analyzed by real-time PCR using BKPyV-specific primers. Error bars indicate standard errors. Statistical significance was determined with a paired, two-tailed *t*-test (GraphPad Prism).

The effect of LY2603618 on virus spread was assessed in RPTECs infected with BKPyV at the reduced multiplicity of 0.1 IU/cell. The virus-infected cell cultures were treated with LY2603618 starting at 1.5 hpi and samples were collected at 3 dpi and 6 dpi. Immunofluorescent staining for TAg showed that there was not a significant difference in the low percentage of TAg-expressing cells at 3 dpi, but Chk1 inhibition markedly reduced the percentage of TAg-expressing cells at 6 dpi (Figure 7A). This reduction in TAg-expressing cells was associated with a decrease in the quantity of BKPyV DNA in the culture medium of LY2603618-treated cells (Figure 7B). Our standard protocol is to introduce LY2603618 at 1.5 hpi, when the viral inoculum is removed and culture medium is added. It is possible that LY2603618 also blocks BKPyV infection in a Chk1-dependent or -independent manner in the first 1.5 hpi. If this additional block were present, it could impact the second and/or third rounds of infection and contribute to the reduction in TAg-positive cells and viral DNA released into the media at 6 dpi. To address this possibility, LY2603618 was added to quiescent RPTECs at 24 h before infection (hbi) and maintained in the media up to 48 hpi, when the cells were fixed. Immunofluorescent analysis of anti-TAg-stained cells revealed that there was no significant difference in percentage of TAg-positive cells in cultures exposed to LY2603618 beginning at 24 hbi compared to cultures treated with LY2603618 beginning at 1.5 hpi (Figure S8). These results indicate that LY2603618 did not block steps essential for the expression of TAg in the first 1.5 hpi.

### 3.7. siRNA-Mediated Knockdown of Chk1 Inhibits Replication of BKPyV DNA

**Figure 8:**
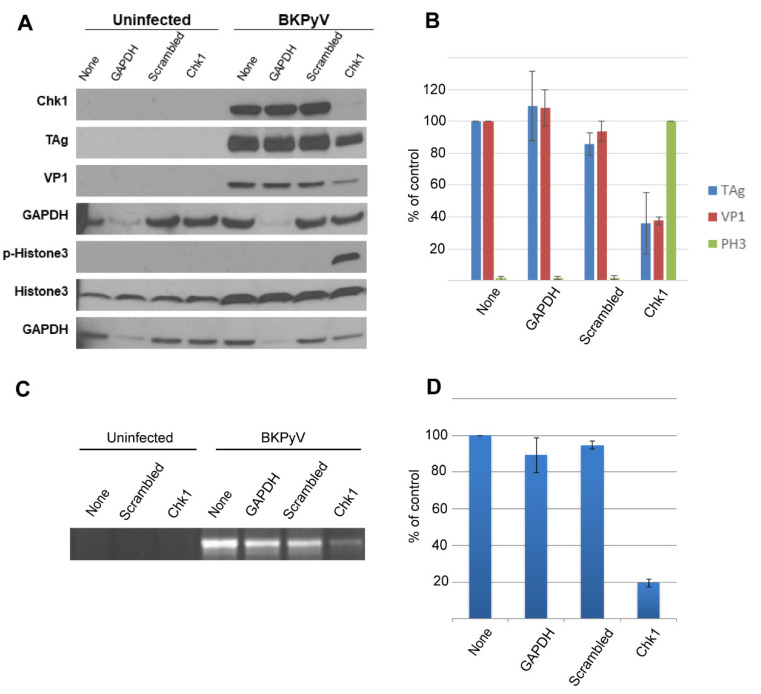
Chk1 knockdown decreases BKPyV proteins and DNA. Confluent RPTECs were made quiescent by removal of growth factors for 2 days. Cells were then transfected with 5 nM Chk1, GAPDH, negative silencer (scrambled), or no (None) siRNA complexes for 24 h. Removal of siRNA complexes was followed by infection with BKPyV Gardner at MOI = 10 for 1.5 h. (**A**) Cells were harvested at 3 dpi by lysis in sample buffer and subjected to immunoblot analysis with the indicated antibodies. (**B**) Protein levels of TAg and VP1 are presented as percent of the untransfected control (None). Levels of pH3-Ser10 (PH3) are relative to the BKPyV-infected, Chk1 knockdown sample; *n* = 3, error bars indicate standard error of the mean. (**C**) BKPyV DNA was isolated from trypsinized cells at 3 dpi and resolved by agarose gel electrophoresis. (**D**) Gels were imaged and ethidium bromide-stained bands were quantified using ImageJ; *n* = 3, error bars indicate standard error of the mean.

To support the finding that LY2603618 was limiting BKPyV replication through inhibition of Chk1, we employed the alternative approach of siRNA-mediated knockdown of Chk1. Confluent RPTECs were deprived of growth factors for two days and transfected with control or Chk1-specific siRNA. Twenty-four hours later, the cultures were infected with BKPyV for 1.5 h and then maintained for 3 days. Immunoblot analysis of whole-cell lysates demonstrated that Chk1 was significantly reduced by the specific Chk1-targeting siRNA, but not GAPDH or scrambled control siRNAs (Figure 8A). Knockdown of Chk1 was associated with decreased expression of viral proteins and stimulation of H3 Ser10 phosphorylation. The 80% reduction in viral DNA in Chk1 siRNA-treated cells (Figure 8B) was similar to the effect of complete Chk1 inhibition by LY2603618 (Figure 5).

## 4. Discussion

### 4.1. BKPyV Infection of Human Renal Epithelium Induces a DNA Damage Response In Vivo and In Vitro

Stimulation of a host cell DDR following polyomavirus infection in vitro is well documented [88]. However, evidence for a DDR following BKPyV infection in vivo is currently limited to increased immunostaining for p53 in PyVAN biopsies [79,89,90]. The phosphorylation of H2AX in BKPyV-infected cells in biopsies provides additional evidence for activation of a DDR in PyVAN. Foci of γH2Ax are considered to be a marker of DNA damage and are known to form in response to DNA double strand breaks (DSBs) and replication stress [50]. The detection of γH2AX in some, but not all TAg-expressing cells in PyVAN biopsies suggests that H2AX is not phosphorylated in all phases of virus infection. γH2AX, visualized by immunoblot and cytometric analyses, was also present in RPTECs infected with BKPyV in vitro. Cytometric data indicated that low levels of γH2AX initially appeared in G1-S phases. Inducible expression of SV40 large T in serum starved cells stimulates appearance of γH2AX [91], showing that expression of large T in the absence of a viral replication origin is sufficient to stimulate phosphorylation of H2AX.

According to our cytometric analyses, BKPyV infection significantly increased levels of γH2AX and TAg in >G2 phase cells, which can reach DNA contents in excess of 10C. This elevated cellular DNA content is consistent with the enlarged TAg-positive nuclei observed in PyVAN biopsies [90]. A similar pattern of increased total DNA content is observed following SV40 infection of monkey kidney cells [92,93,94], where covalently closed, circular SV40 DNA represents 20–30% of the total DNA [95]. These results suggest that both viral and cellular DNA are replicating in >G2 phase of PyV-infected cells. Furthermore, the absence of a gap phase (similar to G1 or G2 phase) in BKPyV-infected cells at or near 8C DNA content is in accordance with rereplication rather than endoreduplication of cellular DNA.

The marked increase in the DDR correlates with progression of infected cells into >G2 phase, where the bulk of viral DNA replication occurs. Based on the patterns of protein phosphorylation, both the ATM and ATR arms of the DDR are activated. It has been shown previously that viral DNA replication is important for activation of ATM and ATR, but the specific stimuli have not been defined [96]. Single-stranded DNA is associated with PML-nuclear bodies in BKPyV-infected cells [97]. Replication protein A (RPA) coats single-stranded DNA, and through associated ATR-interacting protein (ATRIP), stimulates the ATR–Chk1 pathway [98]. Double-stranded DNA breaks arising during replication of viral DNA or rereplication of cellular DNA [99] may be responsible for activation of ATM. Immunoblot analyses demonstrated that both the ATM and ATR arms of the DDR were activated by 2 dpi. Phosphorylation of ATM S1981, Chk2 T68 and Chk1 S317 + S345 decreased after 3 dpi, whereas γH2AX accumulated throughout the time course. Although ATM, ATR and DNA-PK are typically the protein kinases considered responsible for H2AX phosphorylation, γH2AX is retained in BKPyV-infected RPTECs that are triply knocked down for these three kinases [48] indicating that additional kinases may also be involved.

### 4.2. Chk1 Inhibition Restricts Continued >G2 Phase DNA Synthesis

Previous reports of Chk1 inhibition in polyomavirus-infected cells describe a variety of responses: the quantity of BKPyV DNA is reduced by over 60% in infected RPTECs treated with 100 nM UCN-01, a Chk1 inhibitor [48]. Likewise, the amount of a plasmid containing the JCPyV origin of replication is decreased by 50% in IMR32 human fibroblasts co-transfected with a TAg expression vector and cultured in 50 nM UCN-01 [45]. In contrast, SV40 DNA replication in BSC-1 monkey kidney cells, measured by rates of [^3^H]thymidine incorporation, is stimulated by 30% in response to treatment with 100 nM UCN-01 [100]. A third response category is seen with the Chk1 inhibitor PF477736, which has no effect on viral replication or release in mouse embryo fibroblasts infected with murine polyomavirus [53]. These different responses to Chk1 inhibition have not been explained at the molecular level but could result from differences in DDR pathway regulation in specific cell types or in mechanisms of virus replication.

The reports of reduced BKPyV and JCPyV DNA replication in cells treated with Chk1 inhibitors led us to ask whether a clinically-tested and more specific Chk1 inhibitor, such as LY2603618, holds promise as an antiviral agent. Immunoblot analysis established that Chk1 was expressed at low levels in uninfected quiescent RPTECs. A similar pattern is found in serum-starved, quiescent normal human fibroblasts, which express little or no Chk1 mRNA and protein [101]. Serum stimulation results in a rapid increase in Chk1 mRNA and protein in late G1 phase [101]. Following BKPyV infection, total Chk1, pChk1-Ser317 and pChk1-Ser345 were significantly increased by 2–3 dpi. Although mRNA expression in BKPyV-infected cells was not examined, the increase in Chk1 protein is consistent with regulation of the Chk1 gene via a promoter that includes multiple E2F binding sites [102] and may be subject to derepression through TAg binding to retinoblastoma protein family members [103].

Loss of Chk1 protein stability is associated with prolonged replication stress [104], polyubiquitination by the E3 ligases DDB1/Cul4a [105], Fbx6/Cul4a [106] or HUWE1 [107], binding to XIAP/XIAF [108] and absence of binding to ATX3 [109]. A possible role for Chk1 instability in quiescence and in the reduction in Chk1 in BKPyV-infected cells after 3 dpi remains to be explored. In monkey kidney cells lytically infected by SV40, the Mre11-Rad50-Nbs1 (MRN) complex, which is known to assemble at double-strand DNA breaks, is degraded in a proteosome-dependent manner [46]. The decrease in DDR components in both SV40 and BKPyV permissive infection suggests that termination of the checkpoint response may be involved in the virus life cycle. The associated decrease in BKPyV TAg at 6 dpi may result from reduced transcription of the viral early region and/or loss of protein stability. It has recently been shown that BKPyV large T can be targeted for degradation by Skp2 E3 ligase [110].

BKPyV-infected cells treated with a range of LY2603618 concentrations exhibited a concentration-dependent inhibition of Chk1 activity as monitored by Chk1 autophosphorylation at Ser296. In the preclinical characterization of LY2603618, HeLa cells were treated with 100 nM doxorubicin for 24 h to stimulate Chk1 activation and a G2/M phase arrest. Addition of an LY2603618 concentration range to the doxorubicin-treated cells for 2 h allowed determination of an IC50 of 120 nM, as measured by Chk1 S296 autophosphorylation [87]. In a separate experiment, HeLa cells were treated with 125 nM doxorubicin for 24 h, followed by the addition of an LY2603618 concentration range for 7 h. An EC50 of 350 nM for G2/M checkpoint abrogation was determined by measuring the percentage of cells expressing the mitotic marker pH3-Ser10 [87]. However, a higher concentration of LY2603618 (1250 nM) is necessary for generation of aberrant mitotic cells. In general, achieving a Chk1-minus phenotype (incomplete DNA synthesis, damaged DNA and premature mitosis) requires that Chk1 be inhibited by 80%, whereas checkpoint abrogation is achieved at concentrations closer to the IC50 for Chk1 S296 autophosphorylation [87]. Here, we report an IC50 of 640 nM for Chk1 inhibition by LY2603618 in BKPyV-infected RPTECs. This concentration is significantly lower than the LY2603618 concentration of 4 µM required to trigger mitotic events and prevent continued DNA synthesis in >G2 phase. These results indicate that 80–90% inhibition of Chk1 activity is required to induce aberrant mitosis and inhibition of DNA synthesis, both in HeLa cells and in BKPyV-infected RPTECs. In BKPyV-infected RPTECs, this high degree of inhibition can be achieved either by treatment with 4 µM LY2603618 or siRNA-mediated Chk1 knockdown.

A concentration of LY2603618 that inhibited Chk1 activity by 90% limited several interrelated aspects of the viral lytic cycle: first, LY2603618 reduced the percentage of infected cells progressing through >G2 phase. Cells in >G2 phase normally replicate DNA and express both TAg and VP1. The failure to increase total DNA content reflects either DNA synthesis inhibition or cell loss due to cell death and/or detachment. Second, the reduction in the progression of cells through >G phase was associated with a decrease in the expression of TAg and VP1. The decreased quantities of TAg in LY2603618-treated cells appeared to be more marked in immunoblot analysis relative to quantification by LSC. We do not have a definitive explanation for the difference between the techniques. One possibility is that all cells, including floating cells, were analyzed in the immunoblots. LSC only measured cells that remained attached after staining and rinsing. The other explanation is a difference in the access of the TAg antibody to its epitope on immunoblots compared to methanol-fixed cells. Third, the amount of BKPyV DNA was significantly decreased in the absence of Chk1 activity. The decreased quantity of viral DNA in LY2603618-treated cells and Chk1 siRNA transfected cells is consistent with the reduced levels of VP1, which is transcribed and translated after the initiation of viral DNA synthesis [80]. However, our siRNA-mediated Chk1 knockdown experiments did not include cell cycle analysis, so we cannot exclude the possibility that the failure to progress through >G2 phase was an off-target effect of LY2603618. Fourth, in infections initiated at low MOI to monitor virus spread, treatment with LY2603618 until 6 dpi reduced the percentage of TAg-expressing cells and the quantity of viral DNA released into the culture media. Over six days of infection, BKPyV would have undergone several rounds of re-infection. LY2603618 was added after the virus absorption period and again when the cells were fed at 3 dpi. Although some virus is produced in LY2603618-treated cultures, the continued inhibition of Chk1 appears to limit viral DNA replication and possibly virus assembly and release at each round. Pretreatment with LY2603618 starting at 24 hbi and throughout the infection demonstrated that LY2603618 did not block infection, as measured by TAg expression, at steps occurring in the first 1.5 hpi.

### 4.3. Chk1 Inhibition and Stimulation of Mitotic Events

Treatment of BKPyV-infected cells with increasing concentrations of LY2603618 resulted in a corresponding rise in the mitotic marker pH3-Ser10. In time course experiments, pH3-Ser10 was detected at 2 dpi in BKPyV-infected cells exposed to LY2603618 and corresponded to a reduced entry of cells into >G2 phase. Premature activation of mitotic pathways in response to Chk1 depletion or inhibition has been described in a variety of cell lines [111,112]. A similar response is observed in Chk1 inhibition of cell lines delayed in S phase by antimetabolites, except that mitosis is initiated in cells with incompletely replicated DNA [87,113]. The increase in pH3-Ser10 following BKPyV infection was associated with the appearance of aberrant mitotic cells and the failure of infected cells to progress beyond early >G2 phase. The LY2603618-treated cultures also included cells with TAg-positive fragmented nuclei, indicating mitotic catastrophe. This is in agreement with the previous reports of fragmented nuclei in BKPyV-infected RPTECs depleted of ATR, the upstream regulator of Chk1 [48,62] and abnormal mitotic events in SV40-infected CV-1 cells treated with the Chk1 inhibitor UCN01 or the ATM/ATR inhibitor, caffeine [114]. These findings suggest the following model: (1) BKPyV infection stimulates cells into the cycle and holds infected cells in S phase to support continued replication of viral DNA. Host cell DNA also continues to replicate. (2) The regulatory proteins necessary for initiation of mitosis are present but inactive due to intact G2–M phase checkpoint activity that prevents the initiation of mitosis in the presence of ongoing DNA replication. It was previously shown that cyclin B/cdk1, a central factor in triggering mitosis, is present in a tyrosine-phosphorylated, inactive form during SV40 lytic infection [115]. (3) Chk1 inhibition activates mitotic pathways, which drives cells that are actively synthesizing DNA into mitosis. (4) Viral DNA synthesis is reduced due to the absence of replicative DNA synthesis in mitosis and the loss of infected cells through mitotic catastrophe and cell death. In cells that have replicated viral DNA, disruption of virus assembly and release by treatment with LY2603618 is also a possibility. Future studies should focus on the specific pathways downstream of Chk1 that are required for maintenance of viral DNA replication and also any mitosis-independent, direct effects of LY2603618 on viral DNA replication forks.

### 4.4. Chk1 Inhibition as an Antiviral Therapy

Following renal transplantation, antiviral treatments are generally carried out in combination with reduced levels of immunosuppression, which may include antimetabolites such as mycophenolic acid (MPA). MPA inhibits BKPyV replication in vitro in a concentration-dependent manner [116]. This raises the possibility that decreasing the dose of MPA in immunosuppression reduction strategies may contribute to increased BKPyV replication. It will be of interest to determine whether MPA, or other antimetabolites, impact BKPyV replication in combination with LY2603618 or other Chk1 inhibitors.

LY2603618 has been tested in combination with DNA-damaging cancer chemotherapeutics in clinical trials for a variety of cancers. Dosing in these trials generally achieved a C_max_ between 2000 and 4000 ng/mL (equivalent to between 4.5 and 9 µM) [64,67,68,85]. Considering the significant reduction in BKPyV DNA replication in cultured RPTECs at 4 uM LY2603618, it should be feasible to define plasma concentrations sufficient to inhibit BKPyV DNA replication in vivo. Unfortunately, clinical development of LY2603618 for treatment of cancer has been terminated. Increased numbers of thromboembolic events and lack of efficacy were major factors in its discontinuation [65,66]. Other Chk1 inhibitors, tested as single agents or in combination therapies, have also been terminated due to toxicity. Differences in the toxicity profiles of Chk1 inhibitors suggest that some toxicities may be due to off-target effects [117]. In spite of these disappointing results, novel Chk1 inhibitors and Chk1 inhibitors in combination with other therapeutics continue to be a focus of investigation [118]. Clinical trials of Chk1 inhibitors in the treatment of cancer are currently ongoing (clinicaltrials.gov). Inhibitors of other components of the intra-S phase and G2 phase DNA damage checkpoints, such as ATR and Wee1, are also in clinical trials for the treatment of cancer [119]. Similar to our findings with LY2603618, inhibition of ATR or Wee1 kinase activity has been shown to induce mitotic events in BKPyV-infected RPTECs [62]. In addition, Wee1 inhibition resulted in decreased production of viral progeny [62]. These findings suggest that new inhibitors of Chk1, ATR and Wee1, identified in clinical trials as promising anticancer agents, should also be evaluated for possible repurposing as antiviral agents in the treatment of BKPyV disease.

## Data Availability

Data is contained within the article or Supplementary Material.

## Supplementary Materials

The following are available online at https://www.mdpi.com/article/10.3390/v13071353/s1, Figure S1. Comparison of BKPyV Gardner and BKPyV Dunlop stocks by LSC; Figure S2. Uninfected cells in the R7 gate; Figure S3. Cell cycle distribution of BKPyV-infected RPTECs; Figure S4. Viability of uninfected cells treated with LY2603618; Figure S5. LY2603618 inhibits progression of BKPyV-infected cells into >G2 phase; Figure S6. LY2603618 treatment limits replication of BKPyV DNA; Figure S7. Treatment with LY2603618 results in the loss of >G2 phase cells with ongoing DNA synthesis; Figure S8. Early addition of LY2603618 does not block steps essential for TAg expression.
