# Supplementary material for: Chk1 and the Host Cell DNA Damage Response as a Potential Antiviral Target in BK Polyomavirus Infection
Source: Viruses. 2021 Jul 13;13(7):1353. doi: 10.3390/v13071353 (PMC8310304; doi:10.3390/v13071353)
Supplement: Supplementary file 1 [file viruses-13-01353-s001.zip › viruses-1259340-supplementary.pdf]

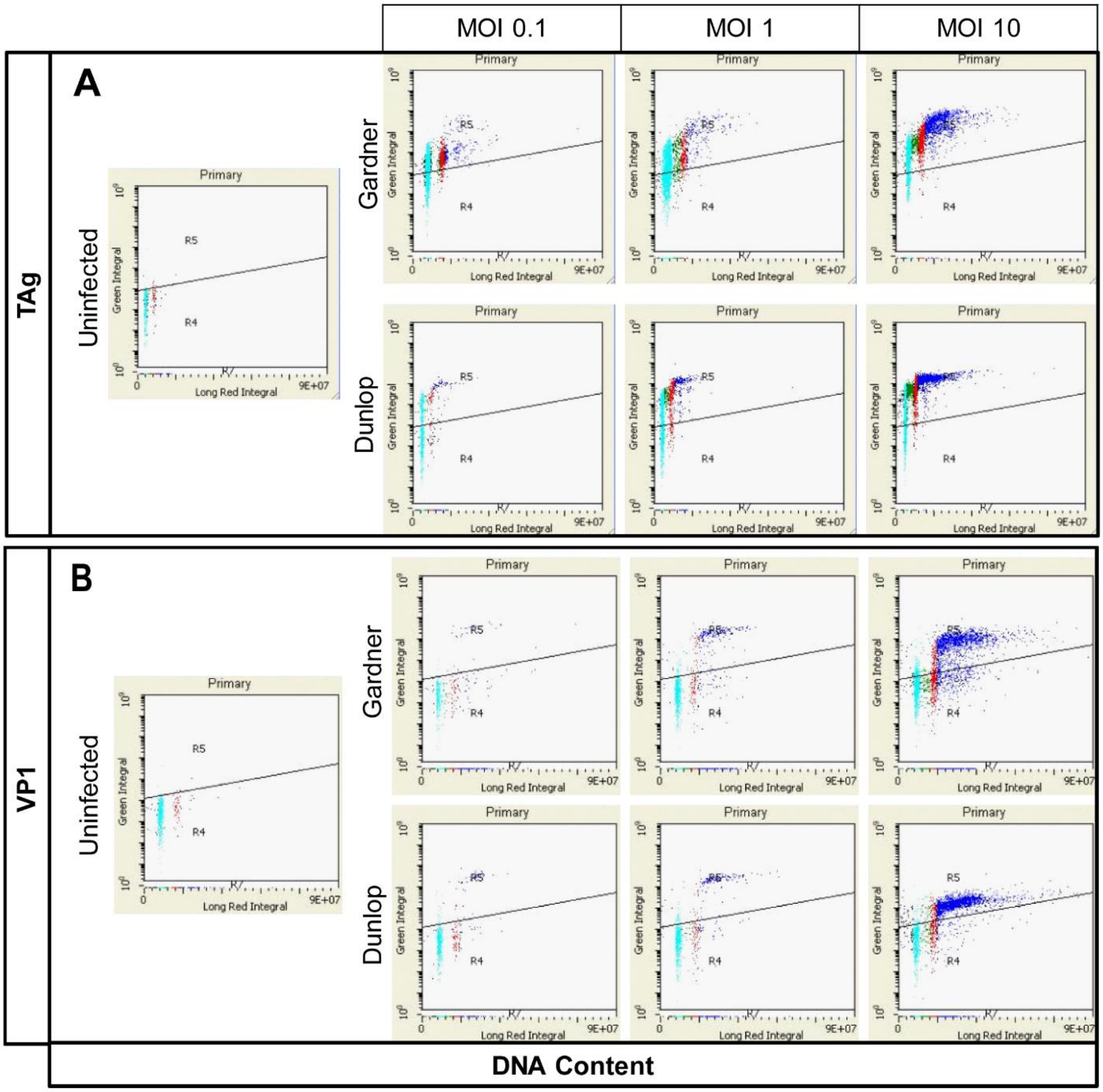

Figure S1. Comparison of BKPyV Gardner and BKPyV Dunlop stocks by LSC. Stocks of Gardner and Dunlop BKPyV were grown in low passage RPTECs and titered on low passage RPTECs by endpoint dilution. Quiescent RPTECs on cover slips were infected with the BKPyV stocks at the indicated MOIs for 1.5 hour. Cells were fixed at 48 hpi, stained with A) anti-TAg (PAb416) and Alexa 488-labeled goat anti-mouse IgG. B) anti-VP1 (PAb597) and Alexa 488-labeled goat anti-mouse IgG. Cover slips were treated with RNase and mounted in propidium iodide prior to LSC analysis.

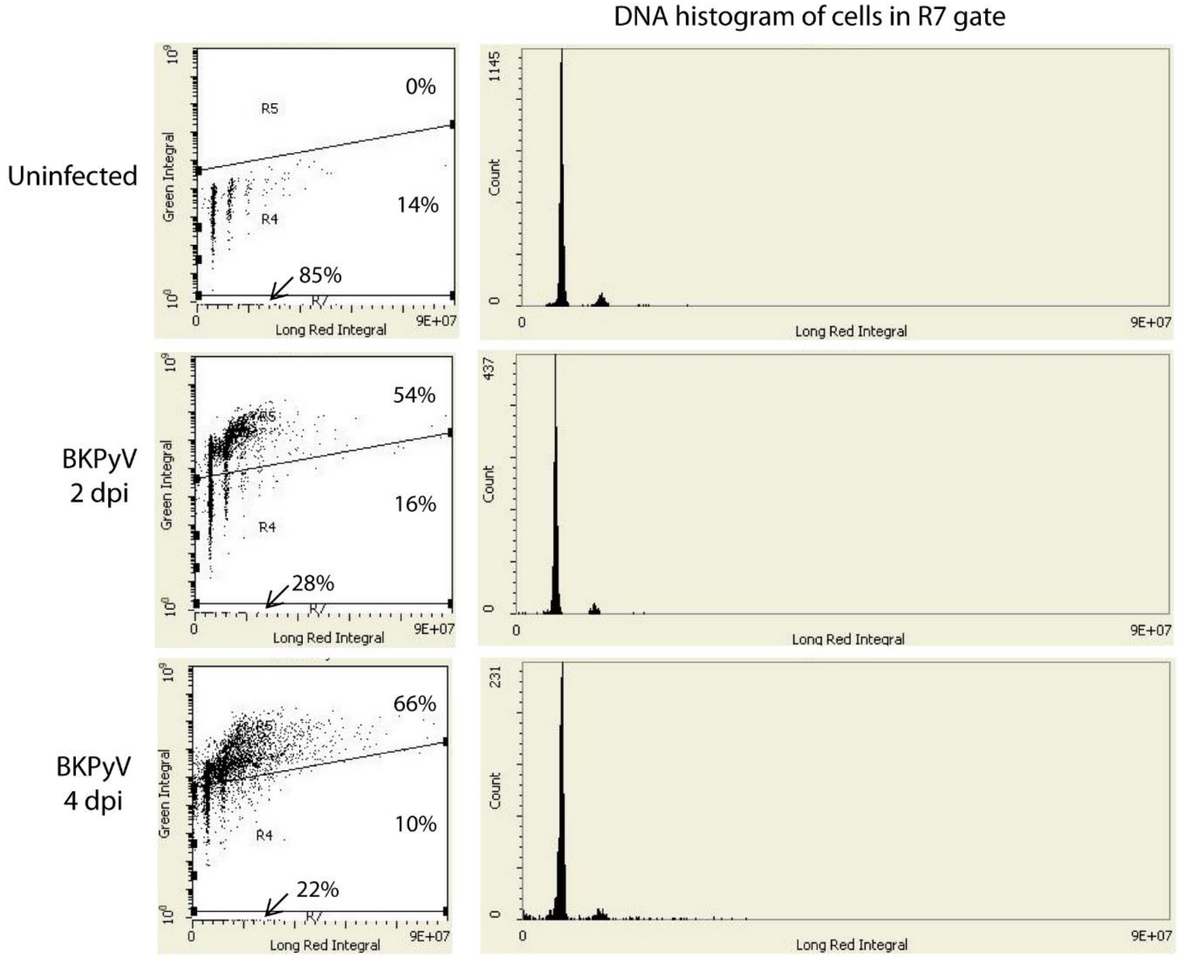

Figure S2. Uninfected cells in the R7 gate. The dot plots shown in the left column were selected from the TAg-stained group in the top row of Figure 3A. Three gates (R4, R5, R7) were set, based on the levels of green fluorescence. Cells with the lowest level of fluorescence fell on the X-axis and were counted in the R7 gate. Expanded DNA histograms of the R7 cells, in the right column, show that the vast majority of cells had a G1 phase DNA content along with a small G2 phase peak.

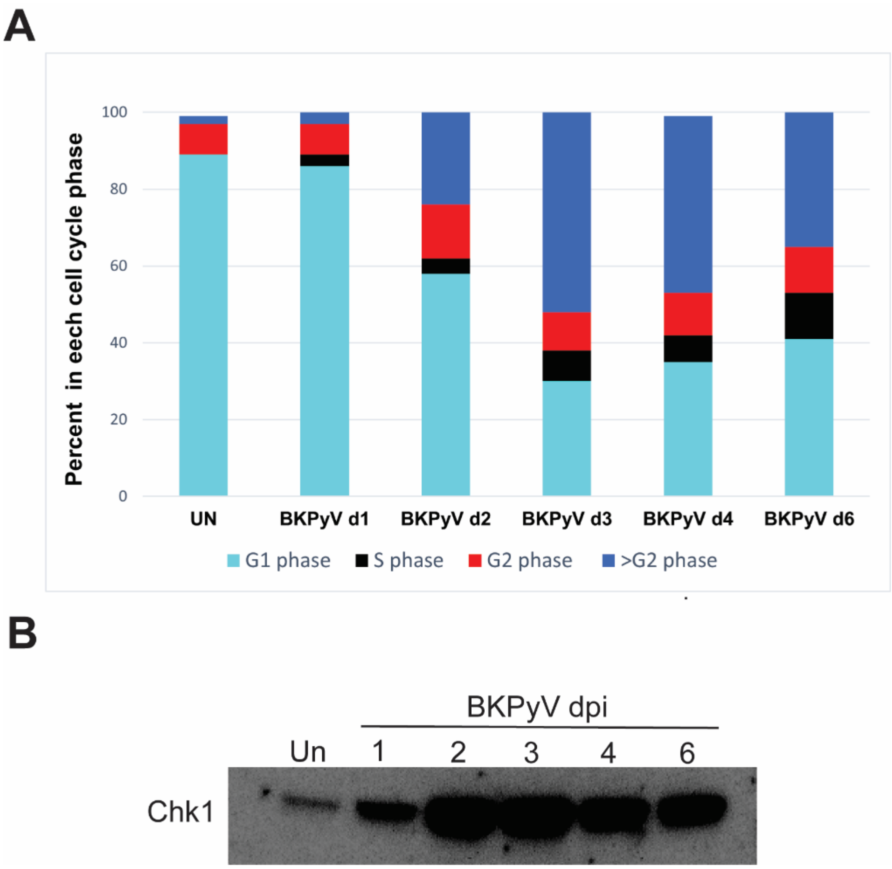

Figure S3. A. Cell cycle distribution of BKPyV-infected RPTECs. The percentages of cells in each cycle phase at increasing times post infection were determined from the cytometric analysis shown in Figure 3A-C. Cell cycle distributions are based on analysis of 15,000 cells at each time point.

B. Overexposed Chk1 immunoblot from Figure 3D shows low levels of Chk1 in uninfected (Un) quiescent cells.

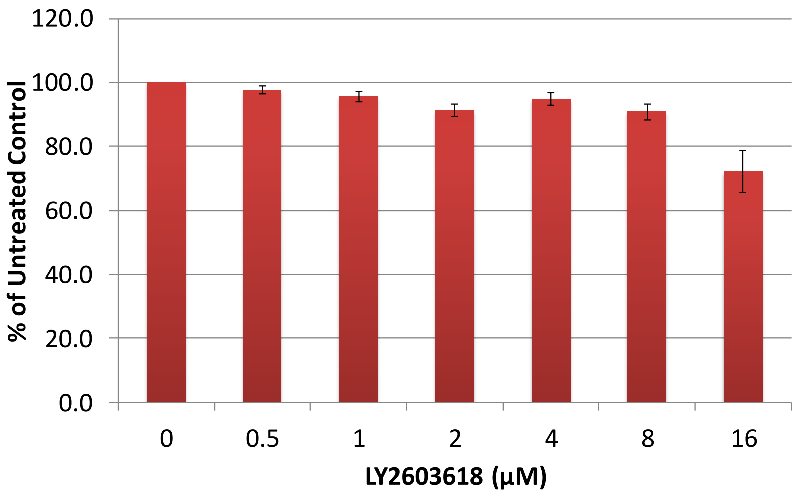

Figure S4. Viability of uninfected RPTECs treated with LY2603618. RPTECs were grown in REGM to near confluence in 96 well tissue culture plates, brought to quiescence in REBM/0.5% FBS and treated with the indicated concentrations of LY2603618. DMSO was at 0.16% in all samples. After 3 days of culture in 37°C/CO_2 incubator, cell viability was measured by the Alamar Blue assay.

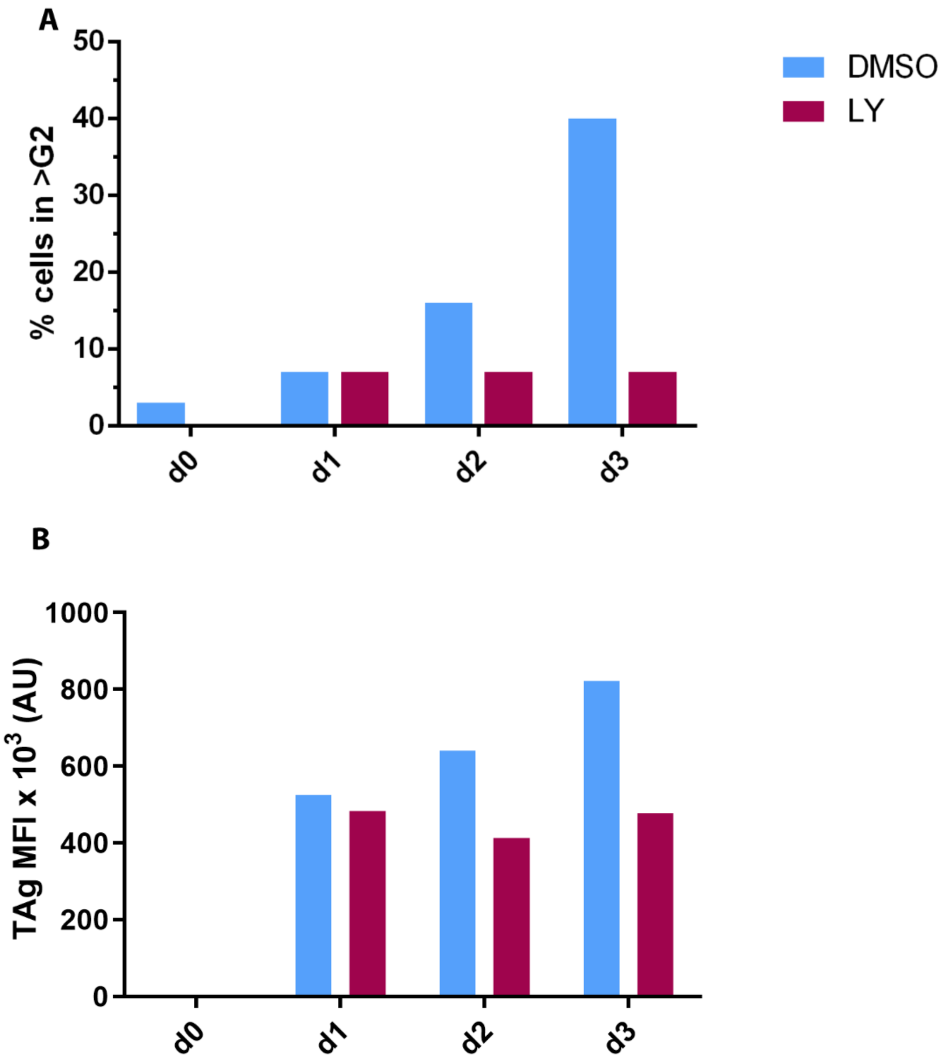

Figure S5. LY2603618 inhibits progression of BKPyV-infected cells into >G2 phase. Data shown is derived from the LSC analysis shown in Figure 5A. A) Addition of 4μM LY2603618 at 1.5 hpi prevents the normal increase in >G2 phase cells following BKPyV-infection. The percentage of >G2 phase cells is based on the total population. B) The mean fluorescence intensity (MFI) of the TAg positive population (gate R5) plateaus in LY2603618-treated cells. The lack of increased TAg expression is best explained by the reduction in >G2 phase cells, which typically express higher levels of TAg

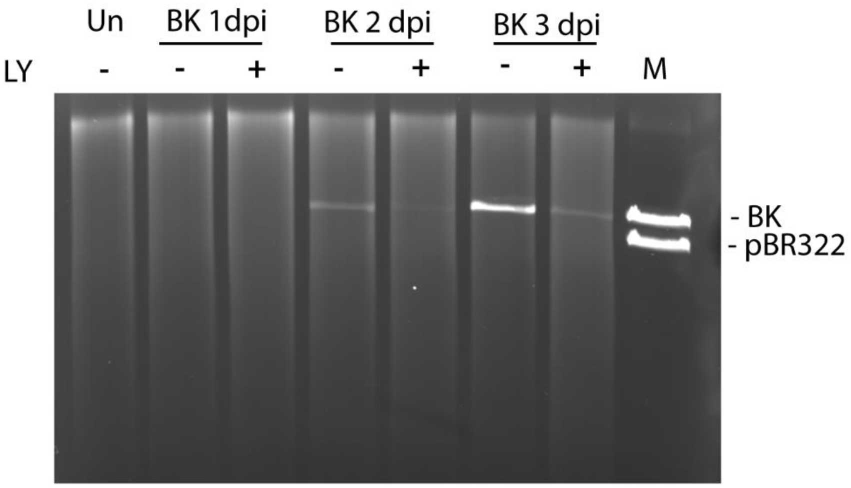

Figure S6. LY2603618 treatment limits replication of BKPyV DNA. Quiescent RPTECs were infected with BKPyV Dunlop (MOI= 10 IU/cell). The inoculum was removed after 1.5 hrs and replaced with REBM/0.5% FBS containing either 4μM LY2603618 (LY) or 0.02% DMSO. At 1, 2 and 3 dpi, attached cells were harvested by trypsinization and pooled with any cells floating in media. Cells were pelleted and frozen at -20°C. DNA was purified from cell pellets using the Qiagen DNeasy Blood & Tissue Kit. 100 ng of DNA was cleaved with BamHI (to linearize BKPyV genomes) and resolved on a 0.6% agarose gel containing ethidium bromide. The marker lane is the plasmid pBKV (34-2) cleaved with BamHI to release the BKPyV genome from pBR322. This analysis differs from the data summarized in Figure 5B in two ways. In this experiment total DNA was purified rather than viral DNA. The background smears in the gel represent BamH1-cleaved cellular DNA. Also, in this experiment the cells were infected with the BKPyV Dunlop strain rather than Gardner used in Figure 5.

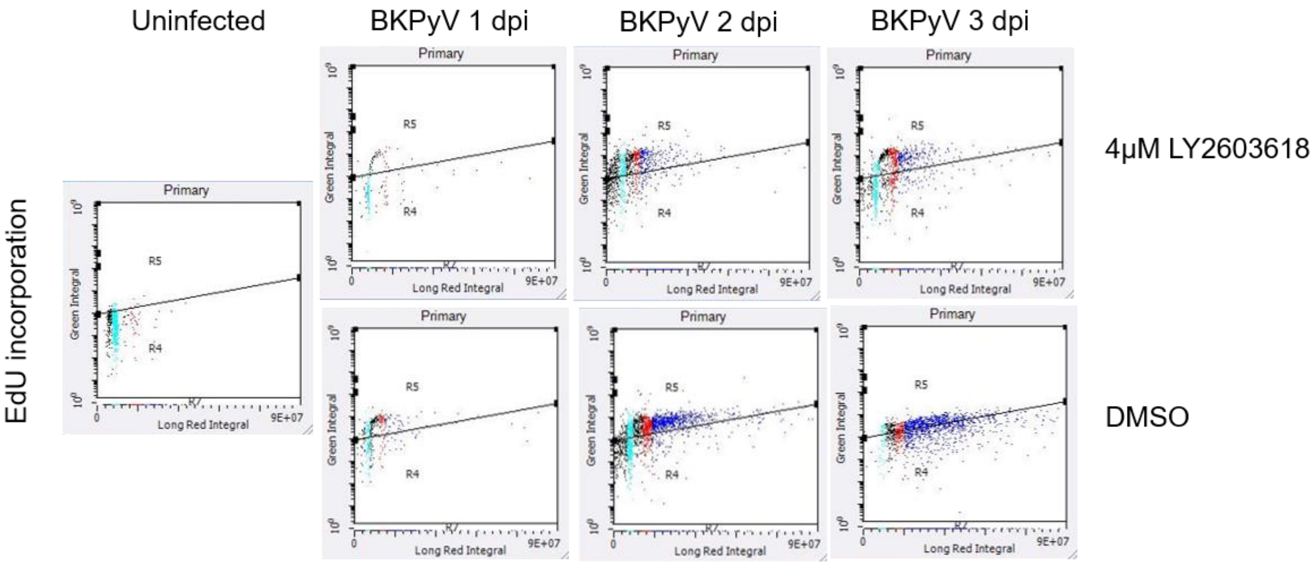

Figure S7. Treatment with LY2603618 results in the loss of >G2 phase cells with ongoing DNA synthesis. Quiescent RPTECs on cover slips were infected with BKPyV Gardner (MOI= 10 IU/cell). The inoculum was removed at 90 min and replaced with REBM/0.5% FBS containing either 4μM LY2603618 or 0.02% DMSO. At 3 dpi, 10 μM EdU, a thymidine analog, was added to the culture media for the final hour prior to fixation of cover slips. Ongoing DNA synthesis was measured by incorporation of EdU. A Click-iT Alexa Flour 488 imaging kit was used to detect EdU. All samples were treated with RNase and stained with propidium iodide prior to LSC analysis.

A

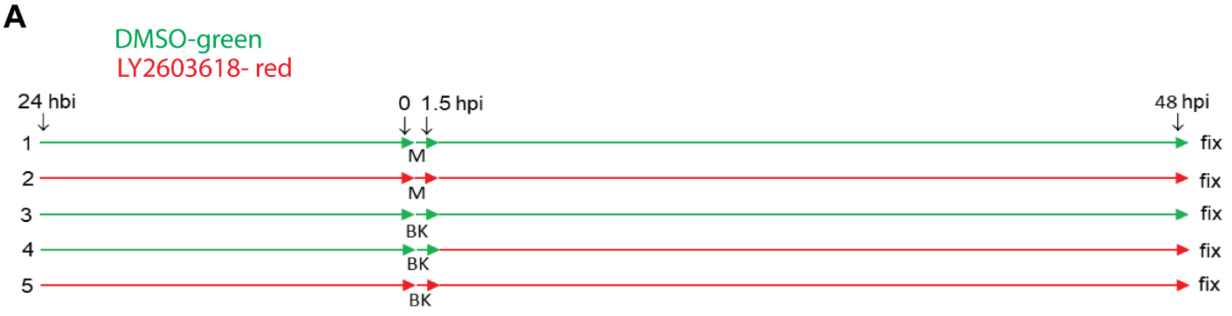

B

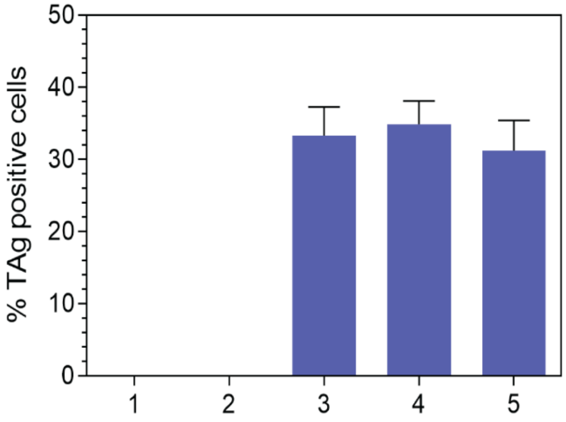

Figure S8. Early addition of LY2603618 does not block steps essential for TAg expression. A) Design of LY2603618-pretreatment time course. Green lines indicate exposure to DMSO and red lines indicate exposure to 4 μM LY2603618. Quiescent RPTECs on coverslips were pretreated for 24 hours before infection (hbi) with either 4 μM LY2603618 (samples 2,5) or 0.02% DMSO (samples 1,3,4). Cultures pretreated with DMSO were then either infected with 1 ml BKPyV Dunlop (BK) (MOI= 10 IU/cell) (samples 3,4) or mock infected with an equal volume of media (M) (sample 1), both containing 0.02% DMSO. Cultures pretreated with LY2603618 were then either infected with BKPyV (sample 5) or exposed to an equal volume of media (M) (sample 2), both containing 4 μM LY2603618 . After a 1.5 hour absorption period, the inocula were removed and replaced with media containing either 4 μM LY2603618 (samples 2,4,5) or 0.02% DMSO (samples 1,3). All coverslips were fixed at 48 hpi. B) Cells were stained with anti-TAg (PAb416), followed by goat anti-mouse Alexa 594. Cover slips were stained with DAPI prior to immunofluorescent microscopy and quantification TAg-positive cells. There were no statistical differences between BKPyV-infection in the absence of LY2603618 and LY2603618 addition at 24 hbi or 1.5 hpi as determined by one way ANOVA (GraphPad Prism). This experiment measured the percentage of TAg-positive cells but did not address the quantities of TAg per cell.
